# Biallelic *NEXN* variants and fetal onset dilated cardiomyopathy: two independent case reports and revision of literature

**DOI:** 10.1186/s13052-024-01678-x

**Published:** 2024-08-26

**Authors:** Irene Picciolli, Angelo Ratti, Berardo Rinaldi, Anwar Baban, Maria Iascone, Gaia Francescato, Alessia Cappelleri, Monia Magliozzi, Antonio Novelli, Giovanni Parlapiano, Anna Maria Colli, Nicola Persico, Stefano Carugo, Fabio Mosca, Maria Francesca Bedeschi

**Affiliations:** 1 Neonatal Intensive Care Unit, Fondazione IRCCS Ca’ Granda Ospedale Maggiore Policlinico, Milan, Italy; 2 Department of Clinical Sciences and Community Health, University of Milan, Milan, Italy; 3 Medical Genetics Unit, Fondazione IRCCS Ca’ Granda Ospedale Maggiore Policlinico, Milan, Italy; 4 Pediatric Cardiology and Arrhythmia/Syncope Units, Bambino Gesù Children Hospital and Research Institute, IRCCS, Rome, Italy; 5 Molecular Genetics Section, Medical Genetics Laboratory, Papa Giovanni XXIII Hospital, Bergamo, Italy; 6 Laboratory of Medical Genetics, Translational Cytogenomics Research Unit, Bambino Gesù Children’s Hospital, IRCCS, Rome, 00165 Italy; 7 Department of Cardio-Thoracic-Vascular Diseases, Fondazione IRCCS Ca’ Granda Ospedale Maggiore Policlinico, Milan, Italy; 8 Fetal Medicine and Surgery Unit, Ospedale Maggiore Policlinico, Fondazione IRCCS Ca’ Granda, Milan, 20122 Italy; 9 Center for Environmental Health, CRC, University of Milan, Milan, 20122 Italy

**Keywords:** Case reports, Dilated cardiomyopathy (DCM), Hypertrophic cardiomyopathy (HCM), Nexilin, *NEXN*

## Abstract

**Background:**

Dilated cardiomyopathy (DCM) is an etiologically heterogeneous group of diseases of the myocardium. With the rapid evolution in laboratory investigations, genetic background is increasingly determined including many genes with variable penetrance and expressivity. Biallelic *NEXN* variants are rare in humans and associated with poor prognosis: fetal and perinatal death or severe DCMs in infants.

**Case presentation:**

We describe two male infants with prenatal diagnosis of dilated cardiomyopathy with impaired ventricular contractility. One of the patients showed hydrops and polyhydramnios. Postnatally, a DCM with severely reduced systolic function was confirmed and required medical treatment. In patient 1, Whole Exome Sequencing (WES) revealed a homozygous *NEXN* variant: c.1156dup (p.Met386fs) while in patient 2 a custom Next Generation Sequencing (NGS) panel revealed the homozygous *NEXN* variant c.1579_1584delp. (Glu527_Glu528del). These *NEXN* variants have not been previously described. Unlike the unfavorable prognosis described for biallelic *NEXN* variants, we observed in both our patients a favorable clinical course over time.

**Conclusion:**

This report might help to broaden the present knowledge regarding *NEXN* biallelic variants and their clinical expression. It might be worthy to consider the inclusion of the *NEXN* gene sequencing in the investigation of pediatric patients with DCM.

## Background

Cardiomyopathies (CMPs) are a heterogeneous group of diseases of the myocardium accounting for the majority of Heart Failure (HF). The most frequent CMPs in adult population are Dilated cardiomyopathy (DCM), whose estimated prevalence varies from 1 in 250 to 1 in 500, and Hypertrophic cardiomyopathy (HCM), ranging from 1 in 500 to 1 in 5000 [1].

Unlike adult population, pediatric CMP is a rare condition that may lead to poor outcomes: nearly 40% of children who present with symptomatic CMP undergo heart transplantation or die within the first 2 years after diagnosis [2].

Pediatric CMP can be an isolated entity or part of complex multisystemic context. Specific cardiac and extracardiac workup must be dedicated to children with CMP due to increased percentage of complex conditions compared to adult onset CMP [3].

Among possible etiologies, genetically determined forms have been increasingly recognized and in recent years significant efforts have been made to unravel the underlying genes: linkage analyses and candidate gene sequencing in familial cases, as well as genome-wide association studies in large cohorts, have contributed to the identification of risk alleles and disease-causing variants, many of which encode for structural components of the cardiac muscle, such as the sarcomere or the cardiac z-disc [4].

*NEXN* encodes the nexilin, an essential protein for Z-disk stability [5, 6]. Few heterozygous pathogenic variants in *NEXN* were found in large cohorts of idiopathic DCM [4] patients and others were reported in pedigrees affected by HCM and their families [7].

The prognosis appears to be worse in those rare individuals carrying biallelic *NEXN* pathogenic variants (either homozygous or compound heterozygous), who usually do not survive childhood [8, 9] or present with lethal form of fetal CMP [10]. We report on two unrelated pediatric cases of *NEXN*-associated CMP, due to biallelic variants, with prolonged survival.

## Case presentation

### Case 1

Patient 1 (P1) was diagnosed with CMP prenatally at 24 weeks of gestation, when the mother (a 38-year-old Egyptian woman) was referred for polyhydramnios, hydrops and severely reduced fetal biventricular systolic function and left ventricular (LV) dilation. Family history revealed parents to be 3rd -degree cousins whereas obstetric history was remarkable for two first-trimester miscarriages and two intrauterine deaths in the late second trimester; the couple also had three healthy children currently in their teens.

**Fig. 1:**
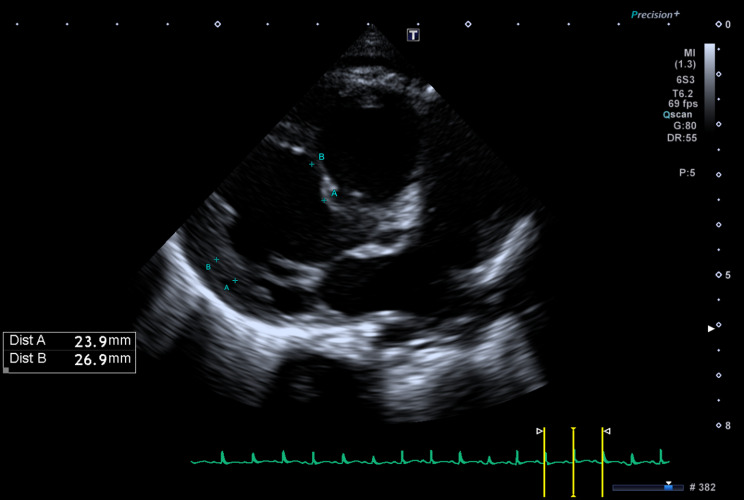
Echocardiogram performed at birth showed biventricular hypertrophy and LV dilation

**Fig. 2:**
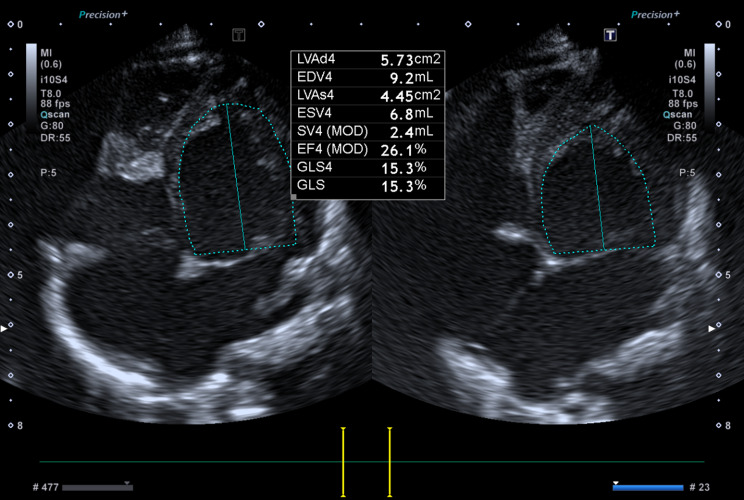
Echocardiogram performed at birth showed severe LV dysfunction with a biplane (Simpson) ejection fraction (LVEF) of 26%

Prenatal ultrasound examination found no evidence of cardiac or extracardiac structural anomalies or sustained arrhythmia to explain myocardial dysfunction; screening for congenital infections (TORCH, Parvovirus, Adenovirus, Coxsackie) was negative except for SARS-CoV-2 IgG and IgM antibodies from an asymptomatic infection. Karyotyping and array-CGH were both normal. The child was born at 34 + 4 weeks of GA by urgent cesarean section, weighing 3240 g (99th percentile). Post-natal echocardiography confirmed the absence of structural heart disease and detected biventricular hypertrophy and LV dilation. LV end-diastolic diameter measured 24 mm (+ 3 z-score) (Fig. 1). The interventricular septum had a peculiar aspect appearing thin, almost membranous and akinetic in the proximal two thirds. Moreover, there was a severe LV dysfunction, as only the apical regions of the cardiac walls were contractile, with a biplane (Simpson) ejection fraction (LVEF) of 26% (Fig. 2). Both atrioventricular valves showed thickened leaflets with preserved mobility and severe regurgitation. No pericardial or pleural effusion were present.

The neonate was started on furosemide and captopril and, because of recurrent episodes of poorly tolerated rapid atrial ectopic tachycardia, on amiodarone. He was initially supported by nasal CPAP and then, upon discharge, he weaned to heated high flow nasal cannula but not to spontaneous ventilation. Extended metabolic screening was performed and was negative.

During the four months of hospitalization, the infant remained in good clinical conditions, echocardiography showed stable cardiac function (LVEF 25–30%) and dimensions. Medical therapy was continued and increased up to the dosage of captopril 0,5 mg/kg every 6 h and furosemide 1 mg/kg every 8 h. Full enteral feeding was reached at three weeks of life, tube feeding was occasionally maintained to prevent the baby’s fatigue. Patient’s growth was regular and satisfactory.

To exclude skeletal muscular involvement, electromyography was undertaken and resulted normal. Diaphragm and intercostal muscles were studied with noninvasive electromyography at different level of noninvasive respiratory support (10 L, 5 L high flow nasal cannula) and in spontaneous breathing and was also normal.

After discharge, at ten months of life, echocardiogram showed improved ventricular function with LVEF 35-40%. He is currently twenty-four months old, he is in good clinical conditions, weaned from respiratory high flow nasal cannula support. Ventricular function at echocardiogram is stable, with good adherence and tolerability to medical therapy. Psychomotor development has been evaluated as adequate.

A trio-based whole exome sequencing (WES) was performed in the patient and both parents [11]. Briefly, the exonic and flanking splice junctions’ regions of the genome were captured using the Clinical Research Exome v.2 kit (Agilent Technologies, Santa Clara, CA). Sequencing was performed on a NextSeq500 Illumina system with 150 bp paired-end reads. Reads were aligned to human genome build GRCh37/UCSC hg19 and analyzed for sequence variants using a custom-developed analysis tool. On average, coverage on target was ≥ 10X for 98% with a mean coverage of 106X. Trio-based WES identified in the proband the homozygous variant c.1156dup (p.Met386fs) in the *NEXN* gene (NM_144573.4); both parents were confirmed to be carriers of the same variant at the heterozygous state. To our knowledge, this *NEXN* variant c.1156dup has never been previously reported and is absent from population database (GnomAD). According to the American College of Medical Genetics and Genomics (ACMG) criteria [12], the variant can be interpreted as likely pathogenic (class 4).

Since the few *NEXN*-heterozygous CMP cases reported in adults developing DCM, HCM or overlapping forms, complete cardiac evaluation was offered to all first-degree relatives. Parents, both heterozygous for the c.1156dup variant, denied personal history of cardiac disease and symptoms of coronary artery disease (CAD) or HF; paternal grandmother died suddenly at the age of 67 for unknown reasons, and a cousin of the mother suffered from Sudden Cardiac Death (SCD) at the age of 47.

The mother, a 38-year-old woman, shows early signs of CMP, consistent with the diagnosis of Hypokinetic non-dilated Cardiomyopathy: left bundle branch block (LBBB) with consequent ventricular dyssynchrony and mildly reduced LVEF (51%), at TTE (TransThoracic Echocardiography) and CMR (Cardiac Magnetic Resonance).

The only cardiac remarkable finding for the father, a man aged 45, is mild LV hypertrophy at TTE. CMR showed normal function (LVEF 57%) and morphology of the left ventricle (EDV 82 ml/mq), with no evidence of edema or fibrosis. Since left ventricular hypertrophy is common in arterial hypertension, we can conclude that the patient’s father does not reach the criteria for possible cardiomyopathy at this time of follow-up. All three asymptomatic siblings aged 13, 15 and 17 years had normal ECG and echocardiograms. Therefore, also considering the absence of affected relatives before adulthood, they have not been tested yet for the familial variant.

### Case 2

Patient 2 (P2) was prenatally diagnosed with DCM at the third trimester of pregnancy. Family history was negative for cardiovascular diseases or recurrent miscarriages. His Italian healthy parents had a remote degree of consanguinity (they both come from a small town in Southern Italy). Prenatal karyotype performed on amniotic fluid revealed a paternally inherited supernumerary marker chromosome, that was also present in the healthy sister.

The patient was born full term by cesarean section. Birth weight was 3600 g (68th percentile), length 51 cm (63th percentile) and OFC 35 cm (62th percentile). Medical treatment for HF was started since birth including diuretics and ACE inhibitors. The patient was referred to our center at the age of 4 years old. Ross functional class was II-III. Echocardiography showed moderate to severe LV dilatation and dysfunction (LVEF 31%), apical LV hypertrabeculation, moderate left atrial dilatation (Vol. 35 ml; 54 ml/m2) and moderate mitral valve regurgitation with tethering of posterior leaflets and focal thickening of the distal part of both mitral leaflets. Medical treatment was modified by adding carvedilol, spironolactone and acetyl salicylic acid. Due to persistent severe LV dysfunction, carvedilol was progressively increased since the age of 5 years old. At the age of 6 years, LVEF increased to 38%. The patient has always shown good adherence and tolerance to therapy. At 8 years old, CMR showed moderate LV dilatation and dysfunction, EDV (end-diastolic volume) 104 ml (indexed 117 ml/m²), ESV (end-systolic volume) 63 ml, systolic ejection 40 ml, FE 39%, mild to moderate mitral insufficiency, normal right ventricular function and dimension. At the age of 10 years, LVEF was 35%, medical therapy was supplemented with ivabradine that was well tolerated. In the following years, a progressive increase in LVEF, which reached 40–45%, was observed. Holter ECG monitoring was always normal. He was never admitted for heart failure. He is currently 15 years-old and has good and stable overall conditions. In addition, the child showed moderate developmental delay and behavioral disturbances.

Next generation sequencing (NGS) analysis was performed at the age of 5 years, on genomic DNA trios by using the Twist Custom Panel (clinical exome Twist Bioscience) on NovaSeq6000 platform (Illumina, San Diego, CA, USA). The reads were aligned to human genome build GRCh37/UCSC hg19. The Dragen Enrichment application of BaseSpace (Illumina) and Geneyx Analysis (knowledge-driven NGS Analysis tool powered by the GeneCards Suite) were used for such mutation for the variant calling and annotation. The coverage on target region was ≥ 10X for 99.4% with a mean coverage of 217.84X. Trio analysis identified the homozygous variant c.1579_1584del p. (Glu527_Glu528del) in *NEXN;* both parents were heterozygous. The variant was evaluated by VarSome (Kopanos et al., Bioinformatics 2018) and classified according to the American College of Medical Genetics and Genomics criteria [14] as of uncertain significance (class3). The Chromosomal Microarray Analysis was performed using the Infinium CytoSNP-850 K BeadChip (SNP-array, Illumina, San Diego, CA, USA), and identified a heterozygous maternal duplication arr[hg19] 12q21.33 (89,683,180 − 89,935,072) x3 of about 251 kb, including (Online Mendelian Inheritance in Man) the disease causing genes *DUSP6* (hypogonadotropic hypogonadism with or without anosmia) and *POC1B* (Cone-rod dystrophy), and classified as of uncertain significance.

As for P1, following the diagnosis, parents underwent cardiac screening and follow-up (every 2–3 years) with normal ECG and echocardiography.

## Discussion and conclusions

*NEXN* is a gene located on human chromosome 1p31.1, encoding for nexilin, a F-acting binding protein abundantly expressed in heart and skeletal muscle, and at lower levels in placenta, lung, liver and pancreas. Hassel et all. described 9 heterozygous forms in adults with DCM and an average onset age of 50 years [13]. In pediatric population a few cases of heterozygous [9, 14, 15] and even rarer cases of biallelic [8–10, 16] *NEXN* variants are reported in literature, and summarized in Table 1. The latter are all associated with very poor prognosis, more often fetal or neonatal death. Rinaldi et al. described two related fetuses with HF, reduced contractility, endocardial fibroelastosis, cardiomegaly and hydrops fetalis, as occurred in P1 here described [16]. Another case described by Bruyndonckx et al. presented with fetal hydrops and died 2 weeks postnatally [9]. Recently Johansson et al. confirm the severity of biallelic loss of function variants in *NEXN* gene, describing a lethal fetal form of DCM, that segregated with a recessive inheritance pattern in a large pedigree of four generation [10].

**Table 1:** Summary of previously reported cases of NEXN variants

Report	NEXN genotype	Prenatal diagnosis (GE)	Prenatal events	Age at onset	CMP type	Medical treatment	End point (death/ VAD/HTx)	Arrhythmic events	Extracardiac features
Klauke et al., 2017	c.1955 A > G (p.Y652C) Heterozygous variant			5 years	Severe DCM	Anti-congestive medication	VAD (22 years)	N.R.	N.R.
Kean et al., 2019	c.1723G > T (p.E575X) + SCN5A mutation Heterozygous variant		Born prematurely 31 weeks	10 weeks	DCM	Anti-congestive medication	Alive (21 years)	N.R.	Monozygotic twin male
	c.1723G > T (p.E575X) + SCN5A mutation Heterozygous variant		Born prematurely 31 weeks	10 weeks	Mild dilation	Anti-congestive medication Dual chamber pacemaker placement	Alive (21 years)	Ventricular arrhythmia	Monozygotic twin male
Al-Hassnan et al., 2020	c.461_462insA (p.Asn154LysfsTer6) Homozygous variant			1 month	DCM	-	Deceased (3 years)	N.R.	N.R.
	c.1171 C > T (p.Arg391Ter) Homozygous variant			1 month	DCM	-	Alive (10 years)	N.R.	N.R.
Rinaldi et al., 2020	c.1756 A > T (p.Lys586Ter) Compound heterozygous variants	DCM, hydrops, (2nd trimester)	IUFD	Prenatal	DCM, endocardial fibroelastosis	N/A	IUFD	N.R.	N.R.
	c.1909_1912del (p.Tyr637AlafsTer48) Compound heterozygous variants	DCM, hydrops, (2nd trimester)	IUFD	Prenatal	DCM, endocardial fibroelastosis	N/A	IUFD	N.R.	N.R.
Bruyndonckx et al., 2021	c.1174 C > T (p.Arg392Ter) Homozygous variant	DCM, hydrops (3nd trimester)	Emergency C-section	Prenatal	DCM, endocardial fibroelastosis	Anti-congestive medication, inotropes, chest drains	Deceased (2 weeks)	N.R.	N.R.
	c.1949_1951del (p.Gly650del) Heterozygous variant			3 months	DCM	Anti-congestive medication	Alive (11 years)	N.R.	N.R.
Johansson et al., 2022	c.1302delG (p.Ile435SerfsTer3) Homozygous variant	DCM (2nd trimester), fetal movements reduction, hydrops	IUFD	Prenatal	DCM	N/A	IUFD	N.R.	N.R.
Current Study Patient 1	c.1156dup (p.Met386fs) Homozigous variant	Polyhydramnios, hydrops, DCM (2nd trimester)		Prenatal	DCM/ HCM	Furosemide, ACEi, amiodarone	Alive (2 years)	Yes	N.R.
Current Study Patient 2	c.1579_1584del (p.Glu527_Glu528del) Homozigous variant	DCM (3rd trimester)		Prenatal	DCM	ACEi, beta-blocker, spironolactone, furosemide and ivabradine	Alive (15 years)	No	Autism spectrum disorder

*AbbreviationsGE* Gestational Age; *CMP* cardiomyopathy; *FGR* fetal growth restriction; *IUFD* intrauterine fetal death; *VAD* Ventricular assist device; *HTx* Heart Transplantation

According to literature, all the heterozygous carriers in the family presented with a DCM of incomplete penetrance and different age-dependent expression. Our patients carry a *NEXN* homozygous variant never described to date. Based on experimental data from *NEXN*-related CMP physiology, both heterozygous and biallelic pathogenic variants lead to nexilin loss-of-function (LoF). While heterozygous variants, mostly non-frameshift deletion or missense, may exert LoF through different mechanisms according to the involved aminoacid residue, all biallelic variants reported so far are frameshift or nonsense, likely resulting in depletion of nexilin transcripts by means of nonsense-mediated decay. Consistently, homozygous nexilin knock-out mice show early-onset progressive DCM [17, 18]. *NEXN*-related CMP with fetal or neonatal onset are highly suggestive for a biallelic form and associated with a poor survival from previous reports. Nevertheless, our patients showed a reduced but stable systolic ventricular function with modest pharmacological treatment, representing the first pediatric individuals with homozygous *NEXN* variant and prolonged survival. Predominant echocardiographic feature in both patients was ventricular dilation, even if mild hypertrophy was also present in P1 and an apical LV hypertrabeculation was observed in P2. It is described that certain additional features may be present in the genetic forms of DCM, including increased LV wall thickness, prominent LV trabeculations and right ventricular or atrial chamber enlargement or dysfunction. These features are important because some DCM genes, such as *NEXN*, have been reported to cause other cardiac phenotypes, which can occasionally overlap in individuals and within families [19]. Furthermore, in P1 early onset arrhythmia was not associated with the presence of other gene alleles that could be responsible for an altered conduction system.

Considering that nexilin is also expressed in skeletal muscle, albeit to a lesser extent, in P1 we studied our patient to rule out skeletal muscle involvement. Electromyography was normal both for upper and lower limb and for respiratory muscle, although a later involvement of skeletal muscles cannot be excluded.

Our cases also emphasize the importance of family history to guide diagnosis. Consanguineity, recurrence of abortions and fetal deaths are important signs of an inheritable heart disease. Likewise, once diagnosis is made, a cardiovascular screening of asymptomatic first-degree family members is mandatory to allow early detection of CMP and improve outcome [20]. As already pointed out by Pinto YM et all. and as observed in the mother of P1, relatives may have more subtle features on ECG or echocardiography that, while not indicating a diagnosis of DCM, may still be relevant. These features include LBBB, LV enlargement, other chamber enlargement, isolated mildly reduced systolic function (LVEF 50–55%), reduced global longitudinal strain, segmental wall motion abnormalities or hyper-trabeculation [21].

Cardiomyopathies (CMPs) are inheritable heart disease characterized by genetic heterogeneity and variable penetrance and expressivity [22]. Multiple genes have been associated with CMPs, and most of these have also been shown to have extensive variation in the normal population. Interpretation of single pathogenic variants from the many thousands of rare polymorphisms present in every individual is challenging. NGS may be useful for clinicians to address diagnosis definition, provide more precise prognostic evaluations and model individualized follow-ups. The availability of genomic data may also suggest genotype-phenotype correlations [23], however, the contextual presence and potential role of other possible genetic or epigenetic factors should be considered for explaining the phenotypic variability and/or the variable clinical evolution of such patients [24].

Our cases show novel homozygous *NEXN* variants associated with onset in fetal life, clinical features of dilated and hypertrophic cardiomyopathy and heart rhythm disturbance with a prolonged survival. Additional individuals with *NEXN*-related DCM are needed to better investigate the pathological mechanisms involved for this gene.

## Data Availability

The datasets used and analysed during the current study are available from the corresponding author on reasonable request.

## Abbreviations

DCM: Dilated Cardiomyopathy
HCM: Hypertrophic Cardiomyopathy
WES: Whole Exome Sequencing
NGS: Next Generation Sequencing
CMPs: Cardiomyopathies
HF: Heart Failure
LV: Left Ventricle
LVEF: Left Ventricle Ejection Fraction
CAD: Coronary artery disease
(LBBB): Left bundle branch block
TTE: TransThoracic Echocardiography
SCD: Sudden Cardiac
CMR: Cardiac Magnetic Resonance
LGE: Late Gadolinium-Enhancement
EDV: End-diastolic volume
ESV: End-systolic volume
OFC: Occipital frontal circumference

## References

[1] Seferović PM, Polovina M, Bauersachs J, Arad M, et al. Heart failure in cardiomyopathies: a position paper from the Heart Failure Association of the European Society of Cardiology. Eur J Heart Fail. 2019;21:553–76. 30989768 10.1002/ejhf.1461

[2] Lipshultz SE, Law YM, Asante-Korang A, Austin ED, Dipchand AI, Everitt MD, Hsu DT, Lin KY, Price JF, Wilkinson JD, Colan SD. Cardiomyopathy in children: classification and diagnosis: a Scientific Statement from the American Heart Association. Circulation. 2019;140(1):e9–68. 31132865 10.1161/CIR.0000000000000682

[3] Lodato V, Parlapiano G, Calì F, Silvetti MS, Adorisio R, Armando M, El Hachem M, Romanzo A, Dionisi-Vici C, Digilio MC, Novelli A, Drago F, Raponi M, Baban A. Cardiomyopathies in children and systemic disorders when is it useful to look beyond the heart? J Cardiovasc Dev Dis. 2022;9(2):47. 35200700 10.3390/jcdd9020047 PMC8877723

[4] Haas J, Frese KS, Peil B, Kloos W, et al. Atlas of the clinical genetics of human dilated cardiomyopathy. Eur Heart J. 2015;36(28):1123–a35. 25163546 10.1093/eurheartj/ehu301

[5] Hassel D, Dahme T, Erdmann J, Meder B, Huge A, Stoll M, Just S, Hess A, Ehlermann P, Weichenhan D, Grimmler M, Liptau H, Hetzer R, Regitz-Zagrosek V, Fisher C, Nürnberg P, Schunkert H, Katus HA, Rottbauer W. Nexilin mutations destabilize cardiac Z-disks and lead to dilated cardiomyopathy. Nat Med. 2009;15(11):1281–8. 19881492 10.1038/nm.2037

[6] Spinozzi S, Liu C, Chen Z, Feng W, Zhang L, Ouyang K, Evans SM, Chen J. Nexilin is necessary for maintaining the transverse-axial tubular system in adult. Circ Heart Fail. 2020;13(7):e006935. 32635769 10.1161/CIRCHEARTFAILURE.120.006935 PMC7583668

[7] Wang H, Li Z, Wang J, Sun K, Cui Q, Song L, Zou Y, Wang X, Liu X, Hui R, Fan Y. Mutations in NEXN, a Z-Disc gene, are Associated with hypertrophic cardiomyopathy. Am J Hum Genet. 2010;87(5):687–93. 20970104 10.1016/j.ajhg.2010.10.002 PMC2978958

[8] Al-Hassnan ZN, Almesned A, Tulbah S, Al-Manea W, Al-Fayyadh M. Identification of a novel homozygous nexn gene mutation in recessively inherited dilated cardiomyopathy. J Saudi Heart Assoc. 2013;25:171–2. 10.1016/j.jsha.2013.03.180

[9] Bruyndonckx L, Vogelzang JL, Bugiani M, Straver B, Kuipers IM, Onland W, Nannenberg EA, Clur SA, Van Der Crabben SN. Childhood onset nexilin dilated cardiomyopathy: a heterozygous and a homozygous case. Am J Med Genet. 2021;185A:2464–70. 10.1002/ajmg.a.62231 PMC8359989 33949776

[10] Johansson J, Frykholm C, Ericson K, Kazamia K, Lindberg A, Mulaiese N, Falk G, Gustafsson PE, Lideus S, Gudmundsson S, Ameur A, Bondeson ML, Wilbe M. Loss of Nexilin function leads to a recessive lethal fetal cardiomyopathy characterized by cardiomegaly and endocardial fibroelastosis. Am J Med Genet A. 2022;188(6):1676–87. 35166435 10.1002/ajmg.a.62685 PMC9306924

[11] Pezzani L, Marchetti D, Cereda A, Caffi LG, Manara, Mamoli D, Pezzoli L, Lincesso AR, Perego L, Pellicioli I, Bonanomi E, Salvoni L, Iascone M. Atypical presentation of pediatric BRAF RASopathy with acute encephalopathy. Am J Med Genet A. 2018;176:2867–71. 30462361 10.1002/ajmg.a.40635

[12] Hershberger RE, Givertz MM, Ho CY, Judge DP, Kantor PF, McBride KL, Morales A, Taylor MRG, Vatta M, Ware S, on behalf of the ACMG Professional Practice and Guidelines Committee. Genetic evaluation of cardiomyopathy: a clinical practice resource of the American College of Medical Genetics and Genomics (ACMG). Genet Med. 2018;20(9):899–909. 29904160 10.1038/s41436-018-0039-z

[13] Hassel D, Dahme T, Erdmann J, Meder B, Huge A, Stoll M, Just S, Hess A, Ehlermann P, Weichenhan D, Grimmler M, Liptau H, Hetzer R, Regitz-Zagrosek V, Fischer C, Nürnberg P, Schunkert H, Katus HA, Rottbauer W. Nexilin mutations destabilize cardiac Z-disks and lead to dilated cardiomyopathy. Nat Med. 2009;15:1281–8. 19881492 10.1038/nm.2037

[14] Kean AC, Helm BM, Vatta M, Ayers MD, Parent JJ, Darragh RK. Clinical characterisation of a novel Scn5A variant associated with progressive malignant arrhythmia and dilated cardiomyopathy. Cardiol Young. 2019;29(10):1257–63. 31477192 10.1017/S1047951119001860

[15] Klauke B, Gaertner-Rommel A, Schulz U, Kassner A, Zu Knyphausen E, Laser T, Kececioglu D, Paluszkiewicz L, Blanz U, Sandica E, Van Den Bogaerdt AJ, Van Tintelen JP, Gummert J, Milting H. High proportion of genetic cases in patients with advanced cardiomyopathy including a novel homozygous plakophilin 2-gene mutation. PLoS ONE. 2017;12(12):e0189489. 29253866 10.1371/journal.pone.0189489 PMC5734774

[16] Rinaldi B, Race V, Corveleyn A, Van Hoof E, Bauters M, Van Den Bogaert K, Denayer E, De Ravel T, Legius E, Baldewijns M, Aertsen M, Lewi L, De Catte L, Breckpot J, Devriendt K. Next-generation sequencing in prenatal setting: some examples of unexpected variant association. Eur J Med Genet. 2020;63(5):103875. 32058062 10.1016/j.ejmg.2020.103875

[17] Aherrahrou Z, Schlossarek S, Stoelting S, Klinger M, Geertz B, Weinberger F, Kessler T, Aherrahrou R, Moreth K, Bekeredjian R, Hrabě de Angelis M, Just S, Rottbauer W, Eschenhagen T, Schunkert H, Carrier L, Erdmann J. Knock-out of nexilin in mice leads to dilated cardiomyopathy and endomyocardial firoelastosis. Basic Res Cardiol. 2016;111(1):6. 26659360 10.1007/s00395-015-0522-5

[18] Liu C, Spinozzi S, Chen JY, Fang X, Feng W, Perkins G, Cattaneo P, Guimarães-Camboa N, Dalton ND, Peterson KL, Wu T, Ouyang K, Fu XD, Evans M, Chen S. Nexilin is a new component of junctional membrane complexes required for cardiac T-Tubule formation. Circulation. 2019;140(1):55–66. 30982350 10.1161/CIRCULATIONAHA.119.039751 PMC6889818

[19] Stacey P, Johnson R, Birch S, Zentner D, Hershberger RE, Fatkin D. Familial dilated cardiomyopathy. Heart Lung Circ. 2020;29(4):566–74. 31974027 10.1016/j.hlc.2019.11.018

[20] Serra G, Antona V, D’Alessandro MM, Maggio MC, Verde V, Corsello G. Novel SCNN1A gene splicing-site mutation causing autosomal recessive pseudohypoaldosteronism type 1 (PHA1) in two Italian patients belonging to the same small town. Ital J Pediatr. 2021;47:138. 34134742 10.1186/s13052-021-01080-x PMC8207710

[21] Pinto YM, Elliott PM, Arbustini E, Adler Y, Anastasakis A, Bohm M, Duboc D, Gimeno J, de Groote P, Imazio M, Heymans S, Klingel K, Komajda M, Limongelli G, Linhart A, Mogensen J, Moon J, Pieper PG, Seferovic PM, Schueler S, Zamorano JL, Caforio ALP, Charron P. Proposal for a revised definition of dilated cardiomyopathy, hypokinetic non-dilated cardiomyopathy, and its implications for clinical practice: a position statement of the ESC working group on myocardial and pericardial diseases. Eur Heart J. 2016;37:1850–8. 26792875 10.1093/eurheartj/ehv727

[22] Hershberger RE, Jordan E, Adam MP, Mirzaa GM, Pagon RA, Wallace SE, Bean LJH, Gripp KW, Amemiya A. Dilated Cardiomyopathy Overv GeneReviews. 2007, PMID: 20301486, Bookshelf ID: NBK1309.

[23] Piro E, Serra G, Antona V, Giuffrè M, Giorgio E, Sirchia F, Schierz IAM, Brusco A, Corsello G. Novel LRPPRC compound heterozygous mutation in a child with early-onset Leigh syndrome french-canadian type: case report of an Italian patient. Ital J Pediatr. 2020;46:140. 32972427 10.1186/s13052-020-00903-7 PMC7517646

[24] Serra G, Antona V, Schierz M, Vecchio D, Piro E, Corsello G. Esophageal atresia and Beckwith-Wiedemann syndrome in one of the naturally conceived discordant newborn twins: first report. Clin Case Rep. 2018;6(2):399–401. 29445485 10.1002/ccr3.1103 PMC5799623

